# Theta Dynamics Contribute to Retrieving Motor Plans after Interruptions in the Primate Premotor Area

**DOI:** 10.1093/texcom/tgab059

**Published:** 2021-10-07

**Authors:** Ryosuke Hosaka, Hidenori Watanabe, Toshi Nakajima, Hajime Mushiake

**Affiliations:** Department of Applied Mathematics, Fukuoka University, Fukuoka 814-0180, Japan; Department of Physiology, Tohoku University School of Medicine, Sendai 980-8575, Japan; Department of Integrative Neuroscience, Faculty of Medicine, University of Toyama, Toyama 930-0194, Japan; Department of Physiology, Tohoku University School of Medicine, Sendai 980-8575, Japan

**Keywords:** action plan, beta oscillation, dorsal premotor area, interrupting task, theta oscillation

## Abstract

To achieve a behavioral goal, we often need to maintain an internal action plan against external interruption and thereafter retrieve the action plan. We recently found that the maintenance and updating of motor plans are reflected by reciprocal changes in the beta and gamma power of the local field potential (LFP) of the primate medial motor areas. In particular, the maintenance of the immediate motor plan is supported by enhanced beta oscillations. However, it is unclear how the brain manages to maintain and retrieve the internal action plan against interruptions. Here, we show that dynamic theta changes contribute to the maintenance of the action plan. Specifically, the power of the theta frequency band (4–10 Hz) of LFPs increased before and during the interruption in the dorsal premotor areas in two monkeys. Without theta enhancement before the interruption, retrieval of the internal action plan was impaired. Theta and beta oscillations showed distinct changes depending on the behavioral context. Our results demonstrate that immediate and suspended motor plans are supported by the beta and theta oscillatory components of LFPs. Motor cortical theta oscillations may contribute to bridging motor plans across behavioral interruptions in a prospective manner.

## Introduction

We often need to cope with an external interruption while performing a sequence of actions that is necessary to achieve an immediate behavioral goal, for example, a telephone call during draft writing, caring for children while cooking, and so on. Such interruptions to the action sequences give rise to conflict or multi-task conditions, requiring a retrieval of the interrupted action in a prospective manner. To cope with the interruptions and successfully retrieve the initial internal action plan, the nervous system needs a mechanism that keeps the action plan intact against the interruption.

When performing a conflict task or multiple tasks, subjects are required to memorize multiple action plans. In the hippocampus, theta oscillations (4–10 Hz) are known to contribute to memory processing (Buzsáki and Draguhn 2004). Hippocampal theta oscillations also emerge when the target is in conflict (Khemka et al. 2017). Moreover, theta oscillations couple the hippocampus and prefrontal cortex (PFC) to support memory integration (Backus et al. 2016). Theta oscillations in the motor cortex have not been well studied from the perspective of memory processing, although there are few reports that have studied theta oscillations in the primary motor cortex (Igarashi et al. 2013; Xu et al. 2019).

Beta oscillations (10–30 Hz) for the primary and premotor cortices have been involved in memory-related functions, such as the preparation of impending motor sets (Sanes and Donoghue 1993; Donoghue et al. 1998) and stable posturing (Baker et al. 1999; Spinks et al. 2008; van Elk et al. 2010). Moreover, the phase of the beta oscillation in the dorsal premotor area (PMd) might code motor planning or memorization of upcoming movements during the delay period (O'Leary and Hatsopoulos 2006; Watanabe et al. 2015). In addition, we showed that beta oscillations in the medial motor areas contribute to the updating and memorization of immediate motor sequences (Hosaka et al. 2016). It is possible that there is a relationship between cortical oscillations and multi-task processing followed by memorization.

In primates, PMd is one of the neural substrates that could be responsible for multi-task processing in the motor domain. Several lines of evidence support this view. Different attributes of a motor plan (e.g., the location of the reaching target and the arm to use) can be integrated into the PMd (Hoshi and Tanji 2000). When monkeys were preparing to reach two targets in an instructed order, a group of PMd neurons showed activity that reflected the selection of the first target, whereas the activity of some others was selective for the second (Shanechi et al. 2012). Furthermore, population neuronal activity in the PMd processes two actions concurrently when either one is possible, and the activity thereafter specifies one of these actions when monkeys are unequivocally instructed on which action to execute (Cisek and Kalaska 2005). Imaging studies of human subjects found that the anterior part of the PMd is more crucial for higher order processing, while movement-related activations are located in the posterior part of the PMd (Picard and Strick 2001; Abe and Hanakawa 2009). The difference between the PMd and ventral premotor cortex (PMv) is that the PMd processes multiple items or multiple information types to achieve actions, while the PMv processes only single information types (Hoshi and Tanji 2002).

To investigate how the brain maintains and retrieves the internal action plan while responding to an interruption, we trained two monkeys to perform a memory-guided motor task with interruptions; these interruptions required the retrieval of the interrupted action by the prospective memory function. The animals were required to perform a movement under visual guidance and thereafter from memory (main task). While the monkey performed the main task from memory, a visual cue that was independent of the main task was presented to serve as an interruption to which the animals had to respond immediately while suspending their action plan for the main task. After this interruption, the animals were required to perform the motor action memorized during the main task. Local field potentials (LFPs) were recorded from the PMd. Our results demonstrate that immediate and suspended action plans are supported by different oscillatory components of LFPs, beta and theta oscillations, respectively.

## Materials and Methods

### Subjects, Health Care, and Animal Procedures

The present study employed two Japanese monkeys (*Macaca fuscata*; L, 8.0 kg and N, 6.0 kg) that were cared for in accordance with the Guiding Principles for the Care and Use of Laboratory Animals of the US National Institutes of Health as well as the Regulations for Animal Experiments and Related Activities at Tohoku University. All animal experiments were approved by the Tohoku University Committee for Animal Experiments (approval no. IDO009–2).

### Behavioral Task

**Figure 1:**
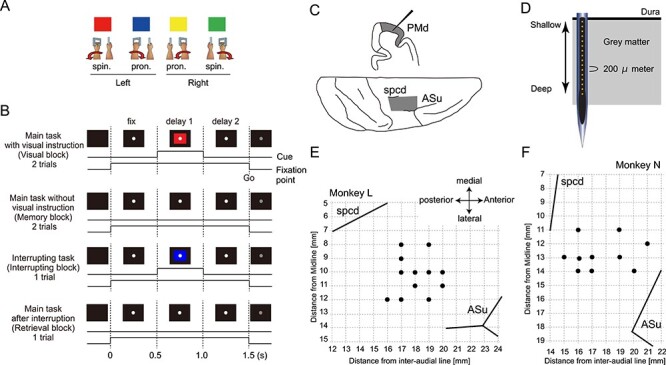
Task sequence and recording sites. (*A*) The associations of colors with movements. The color of a cue indicated the required movement: left forearm supination (red), pronation (blue), right forearm pronation (yellow), or supination (green). (*B*) The temporal sequence of events in each experimental session of the behavioral task. Top panel: main task with visual guidance (visual block). The monkey was required to perform a movement in accordance with the cue. The monkey gazed at the central fixation point on the screen during the initial 500-ms period (fix period). In the next period (delay 1 period), the instruction cue was presented. In the following delay 2 period, the monkey waited for the Go signal for 500-ms. The monkey was required to memorize a particular movement (left forearm supination in this example) while performing the trials two times. Second panel: main task without visual instruction (memory block). The movement was memorized and only the Go signal was given. Third panel: interrupting task (interruption block). The monkey was required to perform a movement in accordance with a cue. The color of the cue was independently determined from the main task. Bottom panel: main task after interruption (retrieval block). The monkey performed the memorized movement of the main task again. (*C*) Schematic diagram of the recording sites. The recording sites in the dorsal premotor area are illustrated in coronal sections obtained at a rostrocaudal level (shaded), as shown on the cortical surface map. Spcd: superior pre-central dimple. ASu: Arculate sulcus. (*D*) Position of the electrode. The electrode was penetrated until the shallowest recording site was located just below the dura mater. The distance between the recording sites was 200 μm. (*E*) Penetration mapping for monkey L. (*F*) Penetration mapping for monkey N.

The animals were seated in a primate chair holding a handle on each hand. We first trained the animals to learn associations of colors with movements (Fig. 1*A*): left forearm supination (red), pronation (blue), right forearm pronation (yellow), and supination (green). The monkeys were required to turn the left or right handle at a deflection angle >5° for each movement. The animals were then trained to perform a movement based on visual cues and thereafter from memory. Finally, the monkeys had to retrieve a motor action in the main task after an interrupting task. Therefore, the behavioral task consisted of four consecutive blocks (Fig. 1*B*). Figure 1*B* illustrates the time course of the experimental session. The temporal structure of the session was as follows:

1. Main task with visual instruction block (visual block): the animals performed a movement in response to colored cues with an intervening delay. The animals had to memorize the main task movement while performing the task. The visual block consisted of two trials.
2. Main task without visual instruction block (memory block): the memorized movement in the main task was repeated without visual guidance. The memory block consisted of two trials.
3. Interrupting block: the animals had to execute a movement (interrupting movement) indicated by a colored cue, branching from the main task. The interrupting movement was independently determined from the main task movement. The interrupting block consisted of one trial.
4. Main task after the interruption block (retrieval block): the animals were required to retrieve and perform the interrupted main task movement once without visual guidance. The retrieval block consisted of one trial. After completion of the retrieval block, a new session was initiated. The movements of the main task and interrupting movement were selected pseudo-randomly. We recorded at least 64 sessions per penetration period.

### Events in Each Trial

At the beginning of the trial, the subjects were required to place the two handles in neutral positions and fixate their eyes on the central fixation point on the screen. They had to hold fixation throughout the trial. For trials of the visual and interrupting blocks, the subjects looked at a central fixation point on the screen during the initial 500 ms period (fix period). Then, a visual cue instructing a movement was presented for 500 ms (delay 1 period). After an additional 500 ms (delay 2 period), the fixation point was dimmed. This served as the movement trigger signal (Go). The animals were required to perform the movement within the reaction time limit (1 s) and then return the handle to the neutral position. Correct movements without a fixation break were rewarded with the delivery of water 500 ms later, which was followed by a 1.5-s inter-trial interval. During the inter-trial interval, the fixation point was not presented on the screen, and eye fixation was not required.

For trials of the memory and retrieval blocks, the subjects looked at a central fixation point on the screen during the initial 1.5-s period. Subsequently, the fixation point was dimmed, which is the movement trigger signal (Go). The monkeys were required to perform memorized movements during the main task. We defined the first 500 ms as the fix period corresponding to the trials of the visual and interrupting blocks. During delay 1, visual cues were not presented for trials of the memory and retrieval blocks.

### Surgery

After 18 months of training, the subjects performed the trials at a correct rate of >80%. Subsequently, an acrylic recording chamber and head-fixation bolts were implanted into the skull of each animal under aseptic conditions using pentobarbital sodium anesthesia (30 mg/kg, intramuscular) with atropine sulphate. Antibiotics and analgesics were used to prevent post-surgical infection and pain, respectively. We localized the PMd based on previously established physiological responses (sensory response profiles and microstimulation effects by a single 1.5 MΩ electrode) (Hoshi and Tanji 2002).

### Recording Electrode and Depth

After complete recovery from surgery, LFPs were recorded in the PMds of both monkeys (Fig. 1*C*,*E*,*F*). For recordings, 16-site linear electrodes (U-probe, Plexon, USA; 185 μm diameter; 200 μm channel separation, Fig. 1*D*) were inserted through the dura mater using a hydraulic microdrive (MO-81, Narishige; Tokyo, Japan). The penetration of the electrode proceeded to the point at which the third shallowest electrode site of the probe detected unit activities and where the most superficial electrode site recorded the physiological LFP waves on the oscilloscope (Fig. 1*D*). Therefore, the deepest site was located 3 mm from the surface of the cortex. The LFPs were band-pass filtered between 0.7 and 300 Hz and recorded at a sampling frequency of 1 kHz. Eye position was monitored using an infrared corneal reflection-monitoring system at 1 kHz (Millennium G200, Matrox, Quebec, Canada).

### Instantaneous LFP Power Calculated by Wavelet Transformation

Customized versions of MATLAB (MathWorks, Massachusetts, USA) and R code were used for the spectral analyses of the LFPs. The time-frequency power of each LFP was obtained using a wavelet transformation with the Gabor mother wavelet as follows:

$$ \begin{array}{l}w\left(t,{f}_0\right)=g(t)\left\{\exp \left(i2{\pi}_0t\right)-\exp \left(-{\sigma}^2{\left(2\pi{f}_0\right)}^2\right)\right\},\\{}g(t)=\frac{1}{2\sigma \sqrt{\pi }}\exp \left(\frac{-{t}^2}{4{\sigma}^2}\right)\end{array} $$

where *t* is time, *f*_0_ is the central frequency, and σ = 5/*f*_0_. The LFP power at the electrode was then calculated by convolving the mother wavelet to the LFP time series.

### Normalizations of LFP Power

The moment-to-moment oscillatory power within each experimental penetration was expressed as a z-score with respect to the mean and standard deviation of the power during the fix periods of the 1st trial of the visual blocks. The frequency resolution of the wavelet transformation was set to 1 Hz.

### Frequency Definition

We defined the frequency bands as 4–10 Hz (theta), 10–30 Hz (beta), 30–80 Hz (low-gamma), and 80–200 Hz (high-gamma). The peak frequency of the beta band for monkeys L and N was 22 and 16 Hz, respectively.

### Epoch-Based Analysis of the Power of theta and beta Bands

To compare the power between trials, we defined epoch-based power as the mean power of each band during the pre-defined period. Specifically, for the theta band, we defined the epoch-of-interest as the fix period, during which theta power typically peaked. For the beta band, we defined the epoch-of-interest as the first 1.5 s from the fixation (from the fix period to the delay 2 period).

### Retrieval-Correct and Retrieval-Error Trials

The conditions of retrieval-correct and retrieval-error were defined as follows: if monkeys successfully performed the movement in the trial of the retrieval block, each correct trial in this session was retrospectively defined as a “trial under the retrieval-correct condition.” Likewise, if monkeys conducted an erroneous choice of movement (incorrect movement) in the retrieval block, each correct trial in this session was retrospectively defined as a “trial under the retrieval-error condition.”

### Current Source Density Analysis

For the laminar analysis, we aligned the LFPs from all electrodes to the middle cortical layer. The middle cortical layer was identified using current source density (CSD) analysis based on the presence of a current sink in response to a visual stimulus (Bastos et al. 2018). In the PMd, an early sink corresponds to the bottom of layer 3 (Godlove et al. 2014), which receives visual sensory inputs (Shipp 2005). CSDs were calculated for the first visual block trials. We then computed the LFP’s second spatial derivative (Mitzdorf, 1985). We subtracted the CSD at the pre-fixation baseline (250 ms to fixation onset) and then z-scored the CSD data over trials by dividing the raw CSD values by their standard error. The CSDs were moving-averaged with a window of 10 ms (Takeuchi et al. 2011). We then assessed which channel (recording site on the electrode) took the minimum CSD value (negative CSD values correspond to current sinks) within a time period from 0 to 1000 ms of fixation onset, and this provided the zero depth (the middle cortical layer) of each penetration.

## Results

### Recording Database

The LFPs were recorded from the PMd while the monkeys performed the interrupting task. Twelve penetrations were conducted in monkey L and ten penetrations in monkey N. These penetrations included 781 sessions in monkey L and 590 sessions in monkey N.

### Dynamic Theta and Beta Power Changes Before and After the Interruption

**Figure 2:**
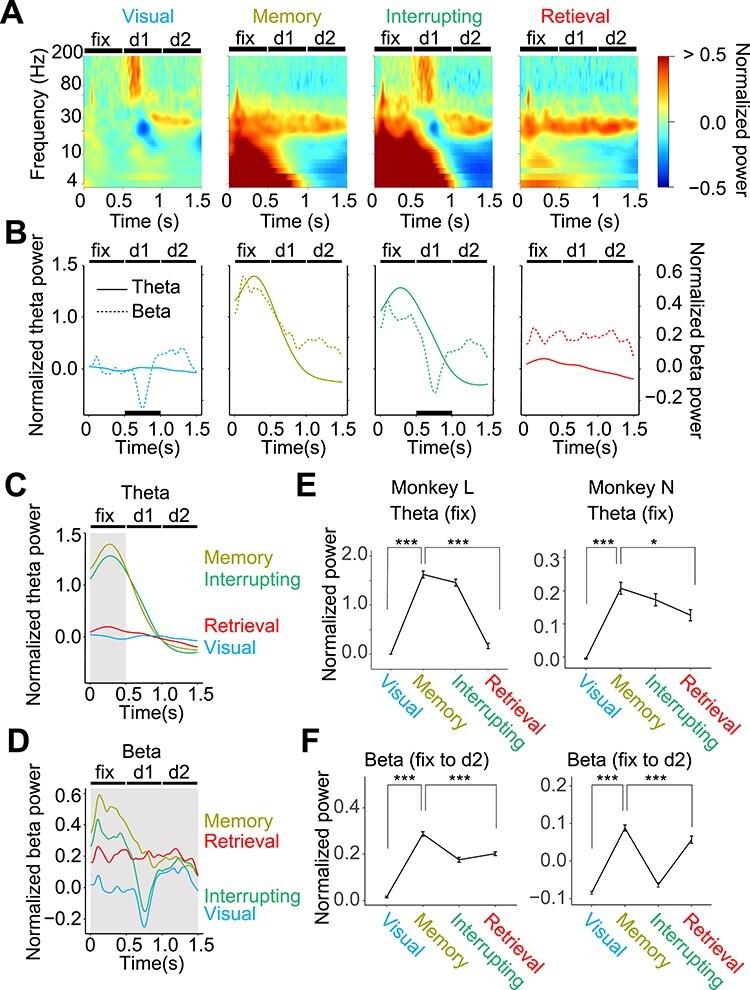
Context-dependent increases and decreases of theta and beta power. (*A*) Time-frequency plot showing the normalized oscillatory power of the LFP recorded in the PMd of monkey L during the visual, memory, interrupting, and retrieval blocks. The frequency (vertical axis) is expressed on a logarithmic scale. The power of each frequency was normalized (z-scored) to the power of the fix period in the first trials of the visual block. D1 and D2 stand for delay 1 and delay 2 periods, respectively. (*B*) Temporal modulation of theta (solid lines, left vertical axis) and beta (dashed lines, right vertical axis) power in the PMd of monkey L. The thick horizontal lines in the visual and interrupting blocks indicate 500-ms intervals for cue presentations. (*C*) The same as in panel *B* but theta power was gathered to compare block differences. The shaded area was used to calculate the mean power in panel *E*. (*D*) The same as in panel *C* for beta power. The shaded areas were used to calculate the mean power in panel *F*. (*E*) The mean power of the theta component (4–10 Hz) during the fix period (shaded area in panel *C*) in the visual, memory, interrupting, and retrieval blocks for monkey L (left) and monkey N (right). (*F*) The mean power of the beta component (21–30 Hz for monkey L and 11–20 Hz for monkey N) during the fix, delay 1, and delay 2 periods (shaded area in panel *D*) for monkey L (left) and monkey N (right). Error bars indicate standard errors. The asterisks indicate comparisons of the mean power between blocks using the Wilcoxon rank-sum test (^*^P < 0.05, ^***^P < 0.005).

We focused on the trial-dependent increase in theta (4–10 Hz) and beta (10–30 Hz) power. Color-coded time-frequency histograms during the fix (0–0.5 s), delay 1 (0.5–1 s), and delay 2 (1–1.5 s) periods, ranging from 4 to 200 Hz in monkey L, are shown in Figure 2*A* (the average across all correct trials). To compare theta and beta power, the temporal modulations of the theta and beta powers of monkey L are shown in Figure 2*B*.

Here, we demonstrate the dynamical change in theta power before and after the interruption, and the difference from that of beta power. In the visual block (Fig. 2*A*,*B*, visual), theta power remained at baseline. Beta power sharply decreased during delay 1 in response to the visual cue; then, it recovered and further increased during the delay 2 period. In the memory block (Fig. 2*A*,*B*, memory), theta power was strongly enhanced during the fix period; then, it decreased and disappeared until the end of the delay 2 period. Beta power also increased during the fix period and kept enhanced moderately during delays 1 and 2. In the interrupting block (Fig. 2*A*,*B*, interrupting), theta power was enhanced during the fix period. The beta power was moderately enhanced during the fix period. In response to the visual cue, beta power sharply decreased during delay 1 and slightly increased during delay 2. In the retrieval block (Fig. 2*A*,*B*, retrieval), theta power decreased1. Beta power was persistently enhanced from the fix to the delay 2 periods.

During cue presentations in the visual and interrupting blocks, the beta power was sharply suppressed. In contrast to beta power, high-gamma (80–200 Hz) power increased during cue presentation (Fig. 2*A*, visual and interrupting blocks). This complementary increase and decrease in beta and high-gamma power appeared to reflect the visual inputs. In a previous study, similar complementary phenomena were found in the medial motor areas, and we concluded that the dynamic change of beta and high-gamma power contributed to the updating and maintenance of movement memory (Hosaka et al. 2015, 2016).

To compare the theta power between blocks, the temporal modulations of theta power are summarized in Figure 2*C*. Theta power was enhanced in the memory and interrupting blocks. During delay 2, theta power was at the baseline level, regardless of the blocks. Theta power did not show a complementary change during cue presentation.

The temporal modulation of the beta power is summarized in Figure 2*D*. Beta power was suppressed (to −0.2) in response to visual cues in the visual and interrupting blocks. The dynamic range of beta power (−0.2–0.6) was smaller than that of theta power (0–1.5).

To test the significance of the changes in theta power, we calculated the mean theta power during the fix period (shaded period in Fig. 2*C*). The mean theta power was the strongest in the memory block (1.60 in monkey L and 0.21 in monkey N, respectively; Fig. 2*E*). The mean theta power in the interrupting block was strong as well (1.42 in monkey L and 0.13 in monkey N).

To do the same for beta power, we calculated the mean beta power from the fix to the delay 2 periods (shaded period in Fig. 2*D*). The mean beta power was the strongest in the memory block (0.29 for monkey L and 0.08 for monkey N, respectively; Fig. 2*F*). The mean beta power in the retrieval block (0.20 for monkey L and 0.06 for monkey N, respectively) was significantly weaker than that in the memory block (*P* < 0.005, Tukey–Kramer test).

### Insufficient Enhancement of theta Power in the Memory Block Leads to Erroneous Retrieval

From the results of the previous section, the enhancement of theta and beta power might correlate with the maintenance of the main task action plan across the interruptions. In the retrieval trials, monkeys made the wrong choice of movement (incorrect movement error) with a high frequency (Table 1). The retrieval error might be due to insufficiency of the encoding or maintenance of the main task action plan. Then, the question arises whether theta and beta power during the memory or interrupting block can predict the performance of the retrieval blocks. If so, the LFP power would be correlated with the successful or erroneous retrieval of the main task. To answer this question, we analyzed the sessions, including erroneous trials in the retrieval block. The number of analyzed trials with and without erroneous performance in the retrieval block was 40 (4.9%) and 783 (95.1%) in monkey L, and 140 (17.6%) and 654 (82.4%) in monkey N, respectively. We compared theta power under retrieval-error and retrieval-correct conditions (see Materials and Methods for the definitions) in the memory, interrupting, and retrieval blocks. Note that the trials in the memory and interrupting blocks were correctly performed in both conditions.

**Table 1:** The number of correct trials, incorrect movement trials, and the ratios

	Visual	Memory	Interrupt	Retrieval
	Monkey L			
# Correct trials	21 848	19 073	10 213	9480
# Incorrect movement trials	87	344	8	243
% Incorrect	0.4	1.8	0.1	2.6
	Monkey N			
# Correct trials	15 218	11 767	7108	5866
# Incorrect movement trials	267	490	265	700
% Incorrect	1.8	4.2	3.7	11.9

Notes: The number of correct trials and incorrect movement trials of all behavioral data in monkey L (upper) and monkey N (lower). “% Incorrect” is the ratio of the incorrect movement trials (the number of incorrect movement trials over the number of correct trials).

**Figure 3:**
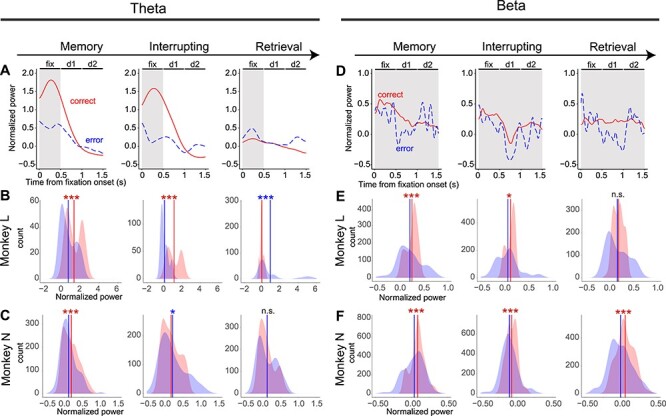
Premature decreases of LFP power in the memory block predict erroneous performance of the retrieval blocks. (*A*–*C*) Theta power in the memory, interrupting, and retrieval blocks under retrieval-correct and retrieval-error conditions. (*A*) Temporal modulations of theta power under retrieval-correct (solid red lines) and retrieval-error (dashed blue lines) conditions (trial average). d1 and d2 stand for delay 1 and delay 2 periods, respectively. The shaded period was used to obtain the mean theta power in panels *B* and *C*. (*B*) The distribution of the mean theta power in retrieval-correct (red) and retrieval-error (blue) conditions in monkey L. The vertical red and blue lines indicate the mean of the distributions. The asterisks indicate comparisons of the means of the distributions using the Wilcoxon signed-rank test (^*^P < 0.05, ^**^P < 0.01, ^***^P < 0.005, n.s. not significant). The red asterisks indicate that the retrieval-correct is greater, and the blue asterisks indicate the opposite. (*C*) The distribution of the mean theta power in monkey N. (*D*–*F*) Beta power. The basic display format is the same as in panels *A*–*C*. Temporal modulation of beta power in panel *D* was fluctuating due to the small number of erroneous trials. The shaded period in panel *D* was used to obtain the mean theta power in panels *E* and *F*.

First, we compared the temporal modulation of theta power under retrieval-correct and retrieval-error conditions in the memory and interrupting blocks (Fig. 3*A*, trial average of monkey L). The peak values of theta power under the retrieval-correct (1.8 in the memory and 1.6 in interrupting blocks) were larger than those under the retrieval-error (0.7 in the memory and 0.65 in the interrupting blocks). In order to statistically quantify the difference, we compared their distribution of the mean theta power (Fig. 3*B* for monkey L and 3C for monkey N). Each mean theta power was calculated at each recording site for each penetration during the fix period. In the memory block, the distribution of the retrieval-correct was biased to positive in monkey L (Fig. 3*B*, left) and its mean (1.65) was significantly larger than the mean of the retrieval-error (0.64, *P* < 0.005, Wilcoxon signed-rank test). This was also true for monkey N (0.21 for correct and 0.13 for error, Fig. 3*C*, left). Therefore, the lack of enhancement of theta power, especially in the memory block, would predict erroneous performance in the retrieval blocks. In the interrupting block, the mean of the distribution of the retrieval-correct condition was larger than that of the retrieval-error condition in monkey L (1.48 for correct and −0.397 for error, Fig. 3*B*, center). However, the relationship was reversed in monkey N (0.18 for correct and 0.21 for error, Fig. 3*C*, center). This subject-depended difference might come from difference in the strategies adapted by two monkeys against interruption under this task. In short, monkey N has tendency to overwrite the movement memory of the suspended main task with the interrupting movement, while monkey L try the random movement to explore possible correct movements when he has less confidence in his memory (see last subsection of the Results section for details).

Subsequently, we assessed whether the decrease in theta power in the retrieval block (see Fig. 2*E*) was correlated with the performance of retrieving the main task. The peak value of theta power was smaller in the retrieval-correct condition (0.23 for correct and 0.5 for error, Fig. 3*A* right panel: average of monkey L). The mean of the distribution of the retrieval-correct (0.16) was significantly smaller than that of the retrieval-error (0.86) in monkey L (*P* < 0.005, Wilcoxon signed-rank test, Fig. 3*B*, right), but it was not significant in monkey N (0.13 for correct and 0.13 for error; Fig. 3*C*, right panel). Therefore, there was a tendency for insufficient suppression of theta power in the retrieval blocks, leading to erroneous performance in the retrieval blocks.

Next, we tested whether beta power similarly predicted the performance of retrieving the main task (Fig. 3*D*–*F*). For the memory block (Fig. 3*E*–*F*, left), the mean of the distribution of beta power under the retrieval-correct condition (0.29 for monkey L and 0.09 for monkey N, respectively) was significantly larger than that of the retrieval-error condition (0.21 for monkey L and −0.04 for monkey N, respectively; *P* < 0.005, Wilcoxon signed-rank test). For the interrupting block (Fig. 3*E*,*F*, center), the mean was larger under the retrieval-correct conditions, and the difference was significant in both monkey L (0.18 for correct and 0.06 for error; *P* < 0.05, Wilcoxon signed-rank test) and monkey N (−0.06 for correct and −0.1 for error; *P* < 0.005, Wilcoxon signed-rank test). In the retrieval block (Fig. 3*E*,*F*, right), the mean was significantly stronger under the retrieval-correct condition in monkey N (0.06 for correct and −0.01 for error; *P* < 0.05, Wilcoxon signed-rank test), but was not significant in monkey L (0.2 for correct and 0.16 for error; Wilcoxon signed-rank test). Therefore, the lack of enhancement of beta power in memory, interrupt, and retrieval blocks would predict erroneous performance in the retrieval blocks.

Taken together, both theta and beta oscillations contributed to predicting performance in the retrieval trial. However, theta power and beta power contribute to the retrieval process after an interruption in a distinct manner. Beta oscillation contributes to correct performance in both the main task and interrupting task movements by enhancing its power. In contrast, theta power showed a biphasic change in the contribution. Specifically, theta power was enhanced in the memory block; subsequently, it decreased in the retrieval block in monkey L and in the interrupting block in monkey N. Moreover, in the retrieval trials, theta power in the correct trials was smaller than that in the error trials.

The retrieval trial was not merely a repetition of the memory trial. Biphasic theta power is specific to the retrieval process. In Supplementary Figure 1, we compare the theta power of correct and error performance in the memory trials. The mean of the distribution of the correct memory trials was larger than that of the error memory trials in both monkeys, although the difference was significant in monkey L (*P* < 0.005, Wilcoxon signed-rank test) and not significant in monkey N (*P* = 0.08, Wilcoxon signed-rank test).

### Area-Dependent Modulation of the LFP Power

**Figure 4:**
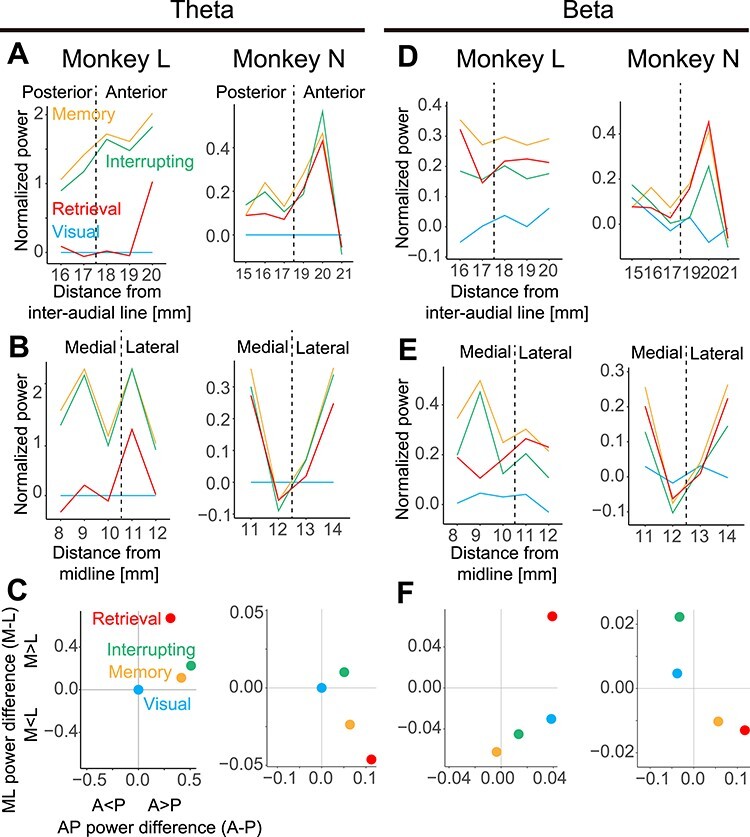
Distributions of LFP power along the anterior–posterior and medio-lateral directions. (*A*) Distribution of theta power in the anterior–posterior direction in monkey L (left) and monkey N (right), during the visual (blue), memory (yellow), interrupting (green), and retrieval (red) blocks. The dashed lines (at 17.5 mm for monkey L and 18 mm for monkey N) were defined as the borders between the anterior and posterior regions of the PMd. (*B*) Distribution of theta power in the medio-lateral direction in monkey L (left) and monkey N (right). The dashed lines (at 10.5 mm for monkey L, and 12.5 mm for monkey N) were defined as the borders between the medial and lateral regions of the PMd (*C*) Distribution of theta power on the AP (anterior–posterior) to ML (medio-lateral) plane. The horizontal axis is the difference of average theta power between the anterior and posterior regions (the posterior is subtracted from the anterior). The vertical axis is the difference of the average theta power between the medial and lateral regions (the lateral is subtracted from the medial). A > P means that average theta power was larger in the anterior than in the posterior. The same applies to A < P, M > L, and M < L. (*D*–*F*) The same as in panels *A*–*C* for beta power.

Previous studies on PMd reported that the anterior part of the PMd is crucial for cognitive functions, and the posterior part is movement related (Fujii et al. 2000; Picard and Strick 2001; Abe and Hanakawa 2009). The motor plan of the main task might be maintained mainly in the anterior part of the PMd. To answer this question, we divided the PMd into four regions (anterior, posterior, medial, and lateral) and compared theta power (Fig. 4*A*–*C*) and beta power (Fig. 4*D*–*F*). In the anterior–posterior direction, theta power was prominent in the anterior PMd region (Fig. 4*A*), while beta power did not show a clear distribution (Fig. 4*D*). In the medial-lateral direction, theta power and beta power did not show a clear distribution (Fig. 4*B*,*E*). To clarify the gradient of theta power across the anterior–posterior axis, we plotted power differences in the anteroposterior and mediolateral directions (Fig. 4*C* for theta, *F* for beta). Theta power was biased toward the anterior-dominant region on the plane. These results are consistent with those of the previous studies.

### Depth Dependent Modulation of LFP Power

**Figure 5:**
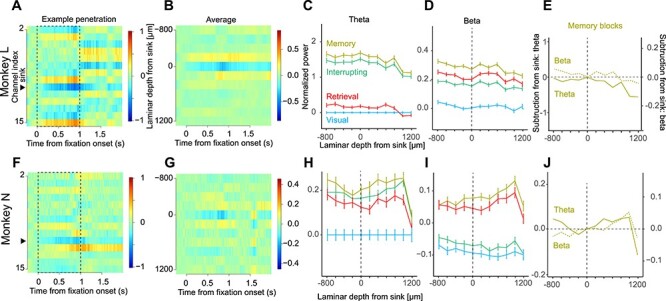
Distribution of LFP power along the laminar direction. (*A*) A current source density analysis of an example penetration in monkey L. Red and blue colors indicate positive and negative CSD values, respectively. The vertical axis is the channel index, and the black triangle indicates the detected sink of this example penetration. The channel that had a minimum CSD value within 0–1000 ms from fixation onset (dashed box) was defined as the sink (zero depth). (*B*) The grand average CSD value in monkey L, calculated after alignment to the detected sink in each penetration. (*C*) Distribution of theta power in the laminar direction in monkey L. (*D*) Distribution of beta power in the laminar direction in monkey L. (*E*) Direct comparison of theta and beta power in the memory blocks. LFP power at the sink was subtracted from the LFP power of each depth. (*F*–*J*) The same as in panels *A*–*E* for monkey N.

Several studies have reported that the LFP power changes with cortical depth (Roopun et al. 2006; Yamawaki et al. 2008; Bastos et al. 2018; Chandrasekaran et al. 2019). To investigate the changes in theta and beta power with cortical depth, LFPs were aligned by the stimulus-dependent sink using the CSD method (Fig. 5*A*, an example penetration for monkey L; Fig. 5*B*, the average for monkey L; Fig. 5*F,G* for monkey N; see Materials and Methods for the calculations), and then the mean theta and beta power of each depth was calculated.

For the theta power of both monkeys (Fig. 5*C* for monkey L and Fig. 5*H* for monkey N), the order of theta power strength (that is, the memory block was the strongest, and the interrupting block was the weakest; see Fig. 2*D*) was preserved regardless of the depth. To investigate if the theta power changes with depth, the power difference from the sink was calculated (i.e., power at the sink was subtracted from the power at each depth). In monkey L (Fig. 5*E*), the deeper the depth, the weaker the theta power. In monkey N (Fig. 5*J*), the deeper the depth, the stronger the theta power.

The same analysis was performed for beta power (Fig. 5*D* for monkey L and Fig. 5*I* for monkey N). In both monkeys, the order of beta power strength was preserved (Fig. 5*D*,*I*). In monkey L (Fig. 5*E*), the deeper the depth, the weaker the beta power. In monkey N (Fig. 5*J*), the deeper the depth, the stronger the beta power.

These results indicate that theta power and beta power were modulated by the laminar in a dependent manner, but this was inconsistent across the two monkeys. The difference of inter-laminar LFP power change might come from the position of the electrode penetration. The penetrations of monkey N were more anterior than that of monkey L (Fig. 1*E*,*F*). It is known that top-down inputs from the PFC flow into deep layers of the PMd. Therefore, one possibility is that LFP power in deep layers is stronger due to the penetration of the electrodes that are biased to the anterior side of the PMd in monkey N.

### Theta Dynamics Difference between Subjects Might Come from Different Strategies against Interruption

**Figure 6:**
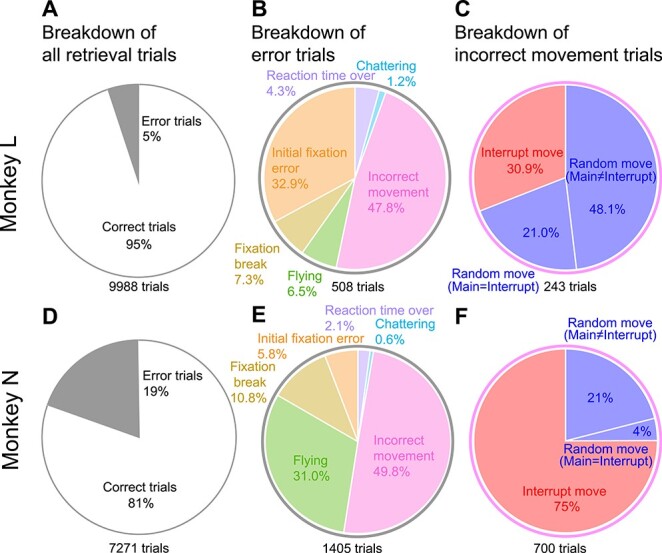
Error movements in the retrieval trials. (*A*) The breakdown of all trials in the retrieval blocks. The white and gray areas are the ratio of correct and error trials, respectively. The number of trials is shown in the bottom of the pie chart. (*B*) Breakdown of the error movements (gray area in panel *A*). The ratio of each error type is shown by the area. (*C*) Breakdown of incorrect movements (pink-colored area in panel *B*). The random movement could be performed in two ways, where the main task movement was the same as the interrupting movement, or it was not. (*D*–*F*) The same as in panels *A*–*C* for monkey N.

In the above results, we found different characteristics in the monkeys. For example, theta power in the retrieval block significantly decreased in monkey L, while it was moderate in monkey N. Low frequency LFP power was strong in the deep layer in one monkey, while it was weak in the other. This individual difference might be due to the difference in strategy against the interruption. To find the strategic difference, we analyzed all behavioral data in the retrieval blocks (Fig. 6). Among 9988 and 7271 trials, the number of the error trials were 508 (5%) for monkey L (Fig. 6*A*) and 1405 (19%) for monkey N (Fig. 6*D*).

**Figure 7:**
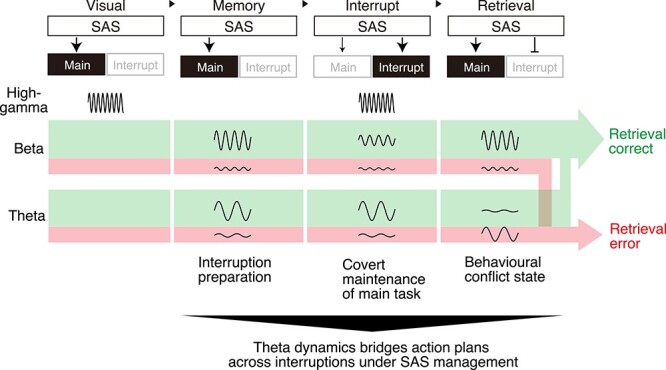
Conceptual scheme of the dorsal premotor cortex under supervisory attentional system control. This scheme describes task-dependent oscillations and their hypothetical functional meanings. Oscillatory components are involved in switching between the single (main task only) and multi-task (main and interrupting tasks) modes of the supervisory attentional system (SAS). The marked arrows in the SAS indicate active maintenance of the action plans. The small pointed arrow in the interrupting block indicates covert maintenance of the main task action plan. The blunt arrow in the retrieval block indicates suppression of the interrupting action. The frequency of each oscillation represents the high-gamma, beta, and theta oscillations. The amplitude of each oscillation reflects the relative magnitude of the high-gamma, beta, and theta oscillations. Green and red colored streams represent the retrieval-correct and retrieval-error conditions, respectively.

In both monkeys, the primary error was “incorrect movement” (47.8% for monkey L, Fig. 6*B*; 49.8% for monkey N, Fig. 6*E*). The incorrect movement is an error where the animals performed movement differed from the movement of the main task. The incorrect movement was composed of two types: the interrupting movement and the random movement. The interrupting movement is erroneously performing the interrupting movement; the random movement is performing neither the main task movement nor the interrupting movement. The majority of the incorrect movements of monkey L was random movements (69.1%, Fig. 6*C*), while the majority in monkey N was interrupting movements (75%, Fig. 6*F*).

From these results, the following can be considered: monkey N might tend to overwrite the movement memory of the suspended main task with the interrupting movement. In contrast, monkey L might tend to try the random movement to explore possible correct movements when he has less confidence in his memory. These strategic differences might be reflected in the individual differences in LFP power.

## Discussion

In the present study, LFPs in the PMd were analyzed to reveal the contribution of increases and decreases in theta and beta power in suspending and retrieving task movements when the main task was interrupted by the visually instructed interrupting task. Our analysis showed that lower-frequency LFP power increased before and during the interruption. Attenuation of theta power before interruption and attenuation of beta power before and during interruption led to erroneous retrieval. Theta and beta power were distinct in retrieval trials: beta power remained enhanced, while theta power recovered to the baseline level. Theta power was more prominent in the anterior part of the PMd. Intra-laminar analysis using CSD revealed a depth dependency under a task condition but inconsistency across the subject. These results suggest that LFP theta power, even in the motor cortex, would contribute to protecting and retrieving the memorized motor plan from an interruption in a prospective manner.

### Why Was the theta Oscillation Observed in the Anterior PMd?

In our findings, theta power in the PMd showed dynamic changes before and after the interruptions: it increased in the memory and interrupting blocks, and decreased in the retrieval blocks. There are few past studies showing theta enhancement in the PMd of behaving animals. A question arises as to why we found the dynamic theta change in the PMd. One possible explanation is the behavioral task. The current behavioral task is a type of the prospective memory task. The prospective memory task requires a high load of cognitive functions to cope with both current and future actions. This may increase theta oscillations in the PMd.

In the current study, the anterior part of the PMd showed larger power than the posterior part of the PMd. The anterior PMd theta oscillation might be affected by theta oscillations of the prefrontal cortex and hippocampus. A large body of evidence demonstrates that theta oscillatory activity in the prefrontal cortex correlates with working memory (Lundqvist et al. 2011; Kornblith et al. 2016; Miller et al. 2018; Holmes et al. 2018), and prospective memory processing recruits large neural circuits, including the PFC. The anterior PMd is strongly interconnected with the prefrontal cortex (Barbas and Pandya 1987; Lu et al. 1994; Picard and Strick 2001; Tanji and Hoshi 2008; Abe and Hanakawa 2009). Moreover, theta oscillations are also generated in the hippocampus, and the hippocampus and PFC are connected via theta oscillations (Fujisawa and Buzsáki 2011). Therefore, both prefrontal and hippocampal neural circuits would utilize theta oscillations for prospective memory processing, and theta oscillations affect anterior PMd theta oscillations.

We also found that theta power recovered to the baseline level in the retrieval blocks, and it remained enhanced in the error trials. In the retrieval trials, the monkeys had to switch from the interrupting task to the main task to retrieve the main task movement, meaning that the monkey was in a conflict state at the retrieval blocks. A previous study reported that hippocampal theta power declines under a conflict state (Sakimoto and Sakata 2014). Therefore, the suppression of theta power in the retrieval block may be elicited by the decline in hippocampal theta power in a conflict state.

### Beta Oscillation Contributes to both Suspended and Immediate Movements

Some findings of the current study regarding beta oscillations are consistent with our past findings in medial motor areas (Hosaka et al. 2016). We found that beta enhancement reflects the maintenance of immediate movement, and found that the lack of beta enhancement led to the erroneous performance of the immediate movement. Moreover, beta power was suppressed in response to cue presentation in the visually guided trials. These results indicate that beta oscillations reflect an immediate state of actions and events, including the maintenance of immediate movement and external events.

Furthermore, beta power before and during the interrupting trials, such as theta power, predicted the performance of the retrieval trials. This indicates that beta oscillations aim to maintain internalized main task movements across trials. Although beta has been widely studied for movement, recent findings also suggest a role in cognitive functions such as working memory (Lundqvist et al. 2016).

Taken together, the dynamic change of the beta oscillation is affected by the cooperation of the internal memory state and external events, while the dynamic change of the theta oscillation depends on the internal memory state.

### Laminar Distributions of LFP Power

In the current study, laminar-dependent LFP power modulation was observed, although it varied among individuals. The laminar distribution of theta and beta power might depend on behavioral contexts or individual differences. This is, to some extent, consistent with those of previous studies. Apical dendrites of principal cells have frequency sensitivity to inputs, indicating the existence of the cortical laminar dependence of LFP amplitude (Watanabe et al. 2014). Several studies have reported that beta power is strong in deep layers or appears only in deep layers, and gamma power is strong in shallow layers (Roopun et al. 2006; Yamawaki et al. 2008; Bastos et al. 2018; Chandrasekaran et al. 2019). In contrast, other studies have reported the opposite phenomenon, where oscillatory power does not differ among different layers (Kondabolu et al. 2016). We tested whether the high-frequency LFP power had an opposite strong-weak relationship from the lower frequency power (Supplementary Fig. 2). The high-gamma power modulation was similar to the lower-frequency power modulation (Supplementary Fig. 2*E*,*J*). Although theta oscillations have been described in the rodent motor cortex (Igarashi et al. 2013), they have not been systematically examined in the non-human primate motor cortex. Further analyses are necessary to clarify the laminar profile of oscillatory power.

### The Prospective Memory and Supervisory Attentional System

The current behavioral task required two types of switching: 1) from the main task to the interrupting task and 2) from the interrupting task to the main task. The current behavioral task could be considered a prospective memory task in the sense that animals are required to retrieve a planned action after the interrupting trial. The prospective memory is a form of memory that involves remembering to perform a planned action or recall a planned intention after a delay (McDaniel and Einstein 2007).

A question arises as to why the PMd is recruited in prospective memory processing. The supporting brain structures of the prospective memory are thought to be the frontal lobe, parietal lobe, hippocampus, thalamus, and cingulate cortices (Okuda et al. 1998; Burgess et al. 2003; Martin et al. 2007; Adda et al. 2008). Among the related areas, the prefrontal cortex (PFC) is related to managing competing behavioral goals and multiple tasks (Norman and Shallice 1986). The PFC might be central to the supervisory attentional system in prospective memory management of other cortical areas (Einstein et al. 2005). Indeed, lateral frontal areas are organized hierarchically from the premotor cortex to the PFC under multi-task conditions (Koechlin et al. 2003). The anterior PMd is strongly interconnected with the PFC (Barbas and Pandya 1987; Lu et al. 1994; Picard and Strick 2001; Tanji and Hoshi 2008; Abe and Hanakawa 2009). Our results suggest that the PMd is recruited in the multi-task processing under the supervisory attentional system of the PFC. The supervisory attentional system can flexibly control other cortical areas, including the premotor area, when the cognitive load is high.

### Summary Scheme of our Findings

To summarize our findings and discussion, we propose a summary schema (Fig. 7). Our scheme consists of three components: the main task, interrupting task, and supervisory attentional system (SAS). The SAS transients in two modes: single task mode (main task only) and multi-task mode (main task and interrupting task).

In the visual trials, the monkeys performed visually instructed movements, and high-gamma power was enhanced to reflect the appearance of visual guidance. In the memory trials, beta power was enhanced, reflecting the main task movement without guidance. Theta power was also enhanced in relation to interruption preparation. In the interrupting trials, high gamma power was enhanced to perform the inserted visually guided movements. Beta and theta power remained enhanced to covertly maintain the main task motor plan as the prospective memory. The lack of enhancement of beta and theta power in the memory and interrupting trials led to erroneous retrieval of the main task movement in the retrieval trials (red stream). In the retrieval trials, beta power was enhanced to perform the retrieved main task movement. In contrast, theta power was suppressed because of the conflict between the two types of memories: the memory of the interrupting action and the memory of the remembered action of the main task. Insufficient enhancement of beta power and insufficient suppression of theta power resulted in an error in the retrieval movement (red stream).

In conclusion, theta oscillations in the PMd are involved in bridging motor plans across behavioral interruptions in a prospective manner under the control of the SAS.

## Supplementary Material

figS1_tgab059

figS2_tgab059

Hosaka_et_al_supplementary_legends_tgab059

## Notes

We thank Ms. M. Takahashi for technical assistance and animal care. *Conflict of Interest*: The authors declare no competing financial interests.
