# Supplementary material for: Theta Dynamics Contribute to Retrieving Motor Plans after Interruptions in the Primate Premotor Area
Source: Cereb Cortex Commun. 2021 Oct 7;2(4):tgab059. doi: 10.1093/texcom/tgab059 (PMC8597970; doi:10.1093/texcom/tgab059)
Supplement: Hosaka_et_al_supplementary_legends_tgab059 [file hosaka_et_al_supplementary_legends_tgab059.docx]

**Figure S1. Error movements in the memory trials.**(A) The breakdown of all behavioural performances in the memory blocks. The white and grey areas represent the ratio of correct and error trials, respectively. The number of trials are shown in the bottom of the pie chart. (B) Breakdown of the error trials (grey area in panel A). (C) Distribution of the mean theta power in correct (red) and incorrect movement (blue) trials in monkey L. The vertical red and blue lines indicate the mean of the distributions. The asterisks indicate comparisons of the means of the distributions using the Wilcoxon signed-rank test (*** p < 0.005, n.s. not significant). (D–F) The same as in panels A–C for monkey N.

**Figure S2. Distribution of LFP power along the laminar direction.**

(A–D, F–I) The same as in Fig. 5A–D, F–I. (E) The distribution of high-gamma power in the laminar direction in monkey L. (J) The same as in panel E for monkey N.
